# Whole blood ratio of *CDK1/CX3CR1* mRNA expression combined to lactate refines the prediction of ICU mortality in septic patients in the Sepsis-3 era: a proof-of-concept study

**DOI:** 10.3389/fmed.2024.1445451

**Published:** 2025-01-03

**Authors:** Marie-Angélique Cazalis, Louis Kreitmann, Guillaume Monneret, Alexandre Pachot, Karen Brengel-Pesce, Jean-François Llitjos

**Affiliations:** ^1^Joint Research Unit HCL-bioMérieux, EA 7426 “Pathophysiology of Injury-Induced Immunosuppression” (Université Claude Bernard Lyon 1 – Hospices Civils de Lyon, bioMérieux), Lyon, France; ^2^Open Innovation and Partnerships (OI&P), bioMérieux S.A., Marcy l’Etoile, France; ^3^Immunology Laboratory, Edouard Herriot Hospital – Hospices Civils de Lyon, Lyon, France; ^4^Department of Anaesthesia and Critical Care Medicine, Hospices Civils de Lyon, Edouard Herriot Hospital, Lyon, France

**Keywords:** sepsis, lactate, intensive care unit, Cdk1, CX3CR1, biomarkers

## Abstract

**Background:**

Transcriptomics biomarkers have been widely used to predict mortality in patients with sepsis. However, the association between mRNA levels and outcomes shows substantial variability over the course of sepsis, limiting their predictive performance. We aimed to: (a) identify and validate an mRNA biomarker signature whose association with all-cause intensive care unit (ICU) mortality is consistent at several timepoints; and (b) evaluate how this mRNA signature could be used in association with lactate levels for predictive and prognostic enrichment in sepsis.

**Methods:**

We conducted a gene expression analysis study at two timepoints (day 1 and day 2–3 following ICU admission) using microarray data from adult septic patients to identify candidate biomarkers predictive of all-cause ICU mortality. We validated mRNA biomarkers using reverse transcriptase quantitative polymerase chain reaction (RT-qPCR) on an external validation cohort. The predictive performance of the mRNA biomarkers combination was assessed in association with lactate level to refine ICU mortality prediction.

**Main results:**

Among 180 chips from 100 septic patients, we identified 39 upregulated and 2 downregulated differentially expressed genes (DEGs) in survivors vs. non-survivors, both at day 1 and days 2–3 following ICU admission. We combined *CDK1*, the hub gene of upregulated DEGs, and *CX3CR1* and *IL1b* to compute expression ratios. The *CDK1/CX3CR1* ratio had the best performance to predict all-cause ICU mortality, with an area under the ROC curve (AUROC) of 0.77 (95% confidence interval [CI] 0.88–0.66) at day 1 and of 0.82 (95% CI 0.91–0.72) at days 2–3 after ICU admission. This performance was better than that of each individual mRNA biomarker. In the external validation cohort, the predictive performance of the *CDK1/CX3CR1* ratio, measured using RT-qPCR, was similar to that of lactate when measure at day 1, and higher when measured at days 2–3. Combining lactate levels and the *CDK1/CX3CR1* ratio, we identify 3 groups of patients with an increasing risk of ICU-mortality, ranging from 9 to 60% with an intermediate-risk group mortality rate of 28%.

**Conclusion:**

With stable predictive performance over the first 3 days following ICU admission, the *CDK1/CX3CR1* ratio identifies three groups of septic patients with increasing ICU mortality risk. In combination with lactate, this novel biomarker strategy may be useful for sepsis patient stratification for personalized medicine trials and ICU management.

## Introduction

Circulating blood lactate is a cornerstone of the diagnosis of septic shock (1). However, despite an unambiguous association between initially and persistently elevated blood lactate values and mortality among septic shock patients, several determinants of this association remain incompletely understood. Increased blood lactate level is believed to reflect an inadequate balance between oxygen demand and supply, and is therefore used as a marker of anaerobic metabolism in the setting of cardiovascular failure. However, several studies have shown that lactic acidosis in sepsis is not solely related to dysoxia (2, 3), which could explain the mixed results of several randomized clinical trials investigating whether lactate guided sepsis resuscitation could improve outcomes in septic patients (4, 5). Recent meta-analyses suggest that lactate management may benefit patients, though further refinement of this approach is necessary (6).

Therefore, the development of relevant and robust biomarkers in sepsis remains a key priority in a precision medicine framework where prognostic and predictive enrichment strategies enable personalized therapies (7, 8). To this end, several biomarkers have been proposed to identify septic patients with poor outcomes apart from lactate (9–15). Regardless of their diagnostic and predictive performances, their validation and rollout as certified diagnostic tools has remained challenging. Difficulties to develop biomarkers in real-world conditions can be linked to methodological biases, but most importantly to the large heterogeneity of patient-related parameters such as clinical presentation, comorbidities, and outcomes. Therefore, some authors have tried to use transcriptomic approaches to take into account this heterogeneity of patients and immune responses (16–20).

Transcriptomic approaches have also been hindered by the substantial variability in the host response to acute injury. For instance, previous transcriptomics studies that have led to the description of distinct sepsis endotypes have shown that around half of patients transitioned from one endotype to another in the first week following ICU admission (21). Contrarily to severe trauma and major surgery, the exact onset of sepsis is rarely known precisely, and therefore the temporal information attached to the values of sepsis biomarkers is often impossible to assess accurately. This might in turn lead to a decreased performance of mRNA biomarkers identified as predictive of outcomes. Thus, the primary aim of this study was to develop an mRNA biomarker combination with stable predictive performance for ICU mortality during the first 3 days following admission. As a secondary objective, we aimed to investigate if this biomarker combination could be associated to lactate levels to improve prognostic enrichment in septic patients.

## Methods

### Discovery cohorts

As a discovery cohort, we retrospectively analyzed gene expression (GE) data from septic shock patients enrolled in three prospective studies using microarray whole-blood transcriptomic analysis performed within the first 72 h after ICU admission [GSE95233 (22), GSE57065 (23) and a third unpublished microarray dataset]. Briefly, all septic shock patients were identified according to the Sepsis-2 diagnostic criteria of the American College of Chest Physicians/Society of Critical Care Medicine (24). Exclusion criteria were age under 18 years and subjects with neutropenia or other immunosuppressive conditions (e.g., HIV infection). The onset of septic shock was defined as the beginning of vasopressor therapy in combination with an identifiable site of infection, persisting hypotension despite appropriate fluid resuscitation and evidence of a systemic inflammatory response manifested by at least two of the following criteria: temperature > 38°C or < 36°C, heart rate > 90/min, respiratory rate > 20/min, white blood cell count >12,000/mm3 or < 4,000/mm3. All patients were part of studies that have been approved by our Institutional Review Board for ethics (“Comité de Protection des Personnes Sud-Est II”), which waived the need for informed consent (#IRB 11236). The third unpublished dataset consisted in 17 additional septic shock patients enrolled in the Immunosepsis study (22).

### Validation cohort

As a validation cohort, we used a multicentric, non-interventional study conducted in 6 ICUs in Lyon from December 2009 to June 2011. Inclusion and exclusion criteria as well as clinical description of the cohort have been published previously (25). Briefly, the patients enrolled were every consecutive patient aged ≥18 years with an expected length of stay in the ICU of more than 2 days. SIRS was defined as the presence of at least two of the following clinical criteria: temperature > 38°C or < 36°C, heart rate > 90 beats/min, respiratory rate > 20 breaths per minute or PaCO2 < 32 mmHg (4.3 kPa), and leucocyte count >12,000/mm3 or < 4,000/mm3 (24). Sepsis was defined as the presence of a proven (visible either clinically/surgically, radiologically, or microbiologically) infection or a highly suspected infection at inclusion. Following the definitions proposed by Vincent et al., which partly rule out the former definitions of sepsis and severe sepsis described in the ACCP/SCCM 1992 consensus conference statement, sepsis was simply defined as an infection requiring ICU admission (26). Shock was defined as persistent hypotension despite adequate fluid resuscitation requiring the use of epinephrine or norepinephrine at a dose >0.25 μg/kg/min (27). Among the patients included in the validation cohort, 4% had sepsis, 38% had severe sepsis, and 58% had septic shock.

### Sample collection, processing, and microarray hybridization

Peripheral blood samples were collected in PAXgene™ Blood RNA tubes (PreAnalytix, Hombrechtikon, Switzerland) to stabilize mRNA. Total RNA was isolated using the PAXgeneTM blood RNA kit (according to the manufacturer’s instructions). Residual genomic DNA was digested using the RNase-Free DNase set (Qiagen Valencia, CA, United States). The integrity of the total RNA was assessed using Agilent 2,100 Bioanalyzer (Agilent Technologies, Santa Clara, CA, United States). Gene expression analysis was conducted using GeneChip® Human Genome U133 Plus 2.0 arrays (Affymetrix, Sta. Clara, CA, United States) according to the manufacturer’s protocol. Raw GE data were normalized using the gcRMA method (28) and technical variability across studies was corrected using the COMBAT algorithm (29).

### Marker selection and evaluation

Differentially expressed genes (DEG) between survivors and non-survivors in ICU were identified using threshold values of the logarithm of fold change expression [log_2_(FC)] >0.6 and q-value <0.1 at two independent time points: during the first 24 h following ICU admission (day 1) and between the 24th and the 72th hour following ICU admission (days 2–3). The intersection of these two gene sets resulted in the final set of genes whose expression was different between survivors and non-survivors in a way that was considered invariable over the first 3 days of ICU stay. On this set of candidate biomarkers, filters were applied to select the final set of mRNA biomarkers: (1) B statistics ≥75% for at least one of the two time points, (2) absolute FC between survivors and non-survivors day 1 and day 2–3 ≥ 1.5 both at day 1 and days 2–3, (3) gene considered as hub genes in pathway analysis (to avoid collinearity), (4) genes whose expression vary in opposite directions (over and under-expressed).

The performance of these mRNA biomarkers to predict all-cause ICU mortality was assessed for each marker individually, and by computing linear and non-linear combinations of these biomarkers. As a performance metric, we computed the area under the receiver operating characteristic (AUROC) curve and its 95% confidence intervals (95%CI), obtained with 2000 bootstrap replicates. We computed sensitivity and specificity values using an optimal threshold calculated by the Youden method (30).

### Platform transfer and RT-qPCR methods

The performance of the *CDK1/CX3CR1* ratio to predict all-cause ICU mortality was then assessed in reverse transcriptase-real time polymerase chain reaction (RT-qPCR) assays using 77 randomly selected mRNA samples from one of the microarray discovery cohort (GSE95233). As advised in the MIQE guidelines (31) to limit the variability of RT-qPCR methods, mRNAs were treated using DNAse and quality was measured using Agilent Bioanalyzer 2,100 (Bio-Rad). Complementary DNAs (cDNAs) were synthesized from the same concentration (200 ng) of total RNA by RT-VILO (Invitrogen) reaction following manufacturer’s instructions. All PCRs were performed in a LightCycler instrument (Roche Diagnostics, Risch-Rotkreuz, Switzerland) using the standard TaqMan Fast Advanced Master Mix PCR kit according to the manufacturer’s instructions (Applied Biosystems, Foster City, CA, United States). Thermocycling was performed in a final volume of 20 μL containing 5 μM of primers and 1 μM of probe. PCR was performed with an initial denaturation step of 10 min at 95°C, followed by 45 cycles of a touch-down PCR protocol (10 s at 95°C, 29 s of annealing at 68°C and a 1-s extension at 72°C). The cDNA standards were prepared from purified PCR amplicons obtained with the corresponding primers. The second derivative maximum method was used with the LightCycler software to automatically determine the crossing point for individual samples, as previously described. Standard curves were generated by using quadruplicate cDNA standard. Relative standard curves describing the PCR efficiency of selected genes were created and used to perform efficiency corrected quantification. Relative fold gene expression was then calculated using the formula: 2^−ΔΔCq^ (32). Additionally, an inter-run calibrator (IRC) was included on each plate in order to obtain relative quantification of each different assay.

### Statistical methods

Categorical variables are reported as number and percentage, and quantitative variables are expressed as median and interquartile range (25-75th percentile). Categorical variables were compared used the chi-square or the Fisher tests, and continuous variables were compared using the non-parametric Mann–Whitney or Kruskal-Wallis tests, as appropriate. Significance levels for *p*-values were set at alph = 0.05 and analyses were two-tailed. Statistical analyses were performed using R (v3.6.2) with packages from the BioConductor library.

Receiver operating characteristic (ROC) curves were computed to assess the accuracy of predictions. ROC curves were compared using the De Long test. We then computed sensitivity, specificity, and positive and negative predictive values using the Youden index. Kaplan–Meier survival curves were used to compare survival between groups, with statistical significance determined using the log-rank test.

The selection of a hub gene was done using three independent nework building databases (Ingenuity Pathway Architect, FunCoup Functional Association network and HumanNet integrated functional network), while taking into account collinearity. The follow-up period of the study was from the time of ICU admission until ICU discharge or death.

## Results

### Microarray discovery cohort

The discovery cohort consisted of 180 microarrays obtained from 100 patients in 3 different septic shock cohorts. Among these, 80 patients had samples at the two time points (day 1 and days 2–3), 4 patients had a sample only at day 1 and 16 patients had a sample only at day 2–3. The main characteristics of patients are summarized in Table 1.

**Table 1:** Patient’s clinical and demographic characteristics in the microarray discovery cohort.

	Total (*n* = 100)	Survivors (*n* = 69)	Non-survivors (*n* = 31)	*p*-value
Age, years	65 (52–75)	65 (52–75)	62 (53–73)	ns
Male gender	64 (64)	46 (67)	18 (58)	ns
Charlson score ≥ 1	46 (46)	31 (45)	15 (48)	ns
SAPS-II score	50 (37–59)	45 (34–55)	60 (47–71)	<0.0001
Infection sites				0.042
Pulmonary	39 (39)	21 (30)	18 (58)	
Abdominal	35 (35)	28 (40)	7 (23)	
Urinary tract	10 (10)	9 (13)	1 (3)	
Others	16 (16)	11 (15)	5 (16)	
Steroids	63 (63)	43 (62)	20 (65)	ns
ICU mortality	31 (31)	0 (0)	31 (100)	

ns, non-significant; SAPS-II, Simplified Acute Physiology Score II; ICU, Intensive care unit. Medians and interquartile ranges [Q1-Q3] are shown for continuous variables or numbers and percentages are presented for categorical variables.

**Figure 1:**
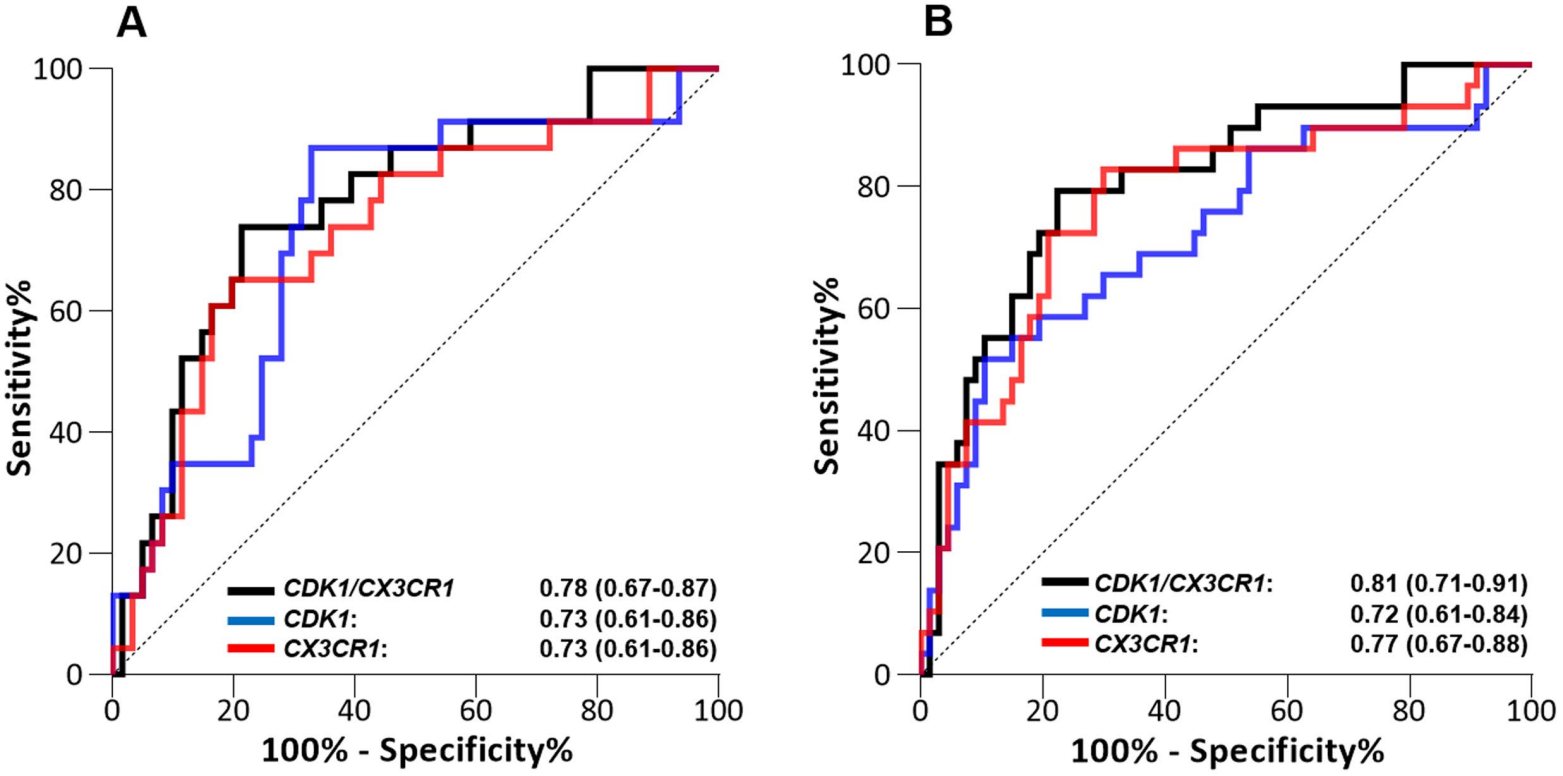
Performance of the *CDK1* gene expression level, *CD3CR1* gene expression level, and *CDK1/CX3CR1* ratio to predict all-cause ICU mortality when assessed on **(A)** day 1 and **(B)** days 2–3 following ICU admission in septic patients (discovery cohort, *n* = 100). Performance is given with area under the ROC curve (AUROC) and its 95% confidence interval.

After co-normalization between microarray cohorts, when comparing survivors to non-survivors, we identified 101 DEGs at day 1, and 173 DEG at days 2–3. Forty-one DEGs were common between the two time points. Among them, only *CX3CR1* and *IL1b* were downregulated in non-survivors, whereas 39 genes were upregulated in non-survivors in comparison to survivors (Supplementary Table 1). Network analysis using three independent network building databases (33, 34) on the 39 remaining genes upregulated in non-survivors identified *CDK1* as the main hub gene. We combined *CDK1* with either *CX3CR1* or *IL1b* to compute expression ratios of up-and down-regulated genes, as this strategy can lift the need to measure the expression of normalization genes. The performance of both individual genes and their ratio to discriminate alive and deceased patients were then computed (Figure 1; Supplementary Figure 1).

The best predictive performance was obtained using the *CDK1/CX3CR1* ratio, with AUROC at day 1 0.78 (95%CI 0.67–0.89) and AUROC at days 2–3 0.81 (95% CI 0.71–0.91). This was better than the predictive performance obtained for each individual marker (AUROC at day 1 0.73 [95% CI 0.61–0.86] and at days 2–3 0.72 [95% CI 0.61–0.84] for CDK1, and AUROC at day 1 0.73 [95%CI 0.61–0.86] and at days 2–3 0.77 [95% CI 0.67–0.86] for *CX3CR1*). The predictive performances of the *CDK1/CX3CR1* ratio was also higher than that of the *CDK1/IL1B* ratio at both time points.

### RT-qPCR platform transfer

To assess the robustness of our results, the performance of the *CDK1/CX3CR1* ratio was then assessed in RT-qPCR using 77 randomly selected mRNA samples from one of the microarray discovery cohort (GSE95233). We found that after platform transfer, the *CDK1/CX3CR1* ratio retained good predictive performance at both day 1 (AUROC 0.83 [95%CI 0.64–1.0]) and days 2–3 (AUROC 0.75 [95%CI 0.59–0.91]). Using the Youden Index, calculated from the qRT-PCR data, the optimal threshold value of *CDK1/CX3CR1 ratio* to distinguish between survivors and non-survivors was 0.47. This value was applied in the external and independent cohort.

### Predictive performance using RT-qPCR on the validation cohort

**Figure 2:**
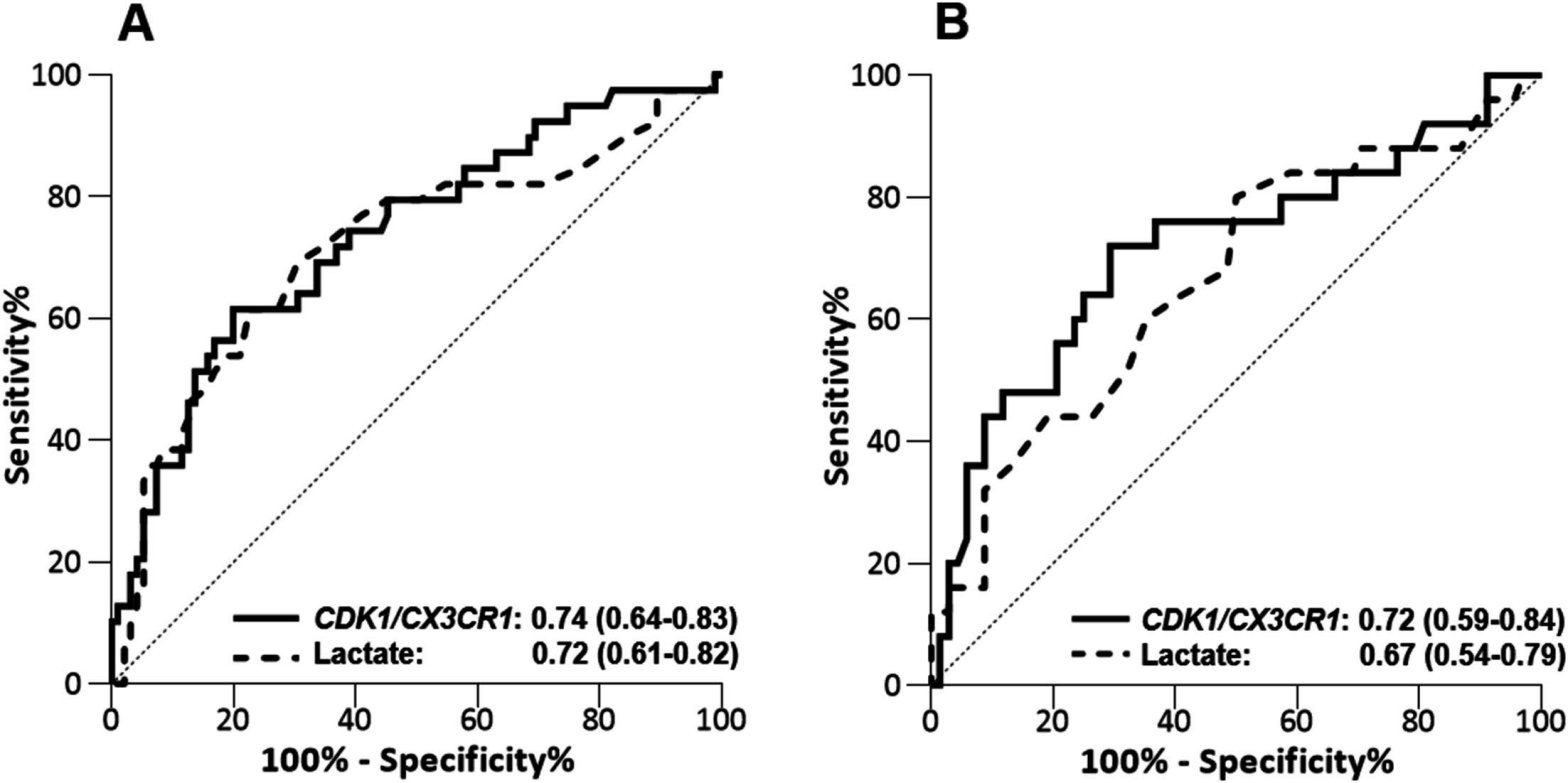
Performance of CDK1/CX3CR1 ratio assesed by RT-qPCR and lactate levels to predict death in the ICU at day 1 **(A)** and days 2–3 **(B)** in the validation cohort (*n =* 140). Performance is given with area under the ROC curve (AUROC) and its 95% confidence interval.

The validation cohort consisted in an independent cohort of 140 septic ICU patients, whose main characteristics are summarized in Table 2 (25). Among them, 134 had a lactate measurement performed at ICU admission, and these patients were selection for assessing the *CDK1/CX3CR1* ratio. The predictive performance of the *CDK1/CX3CR1* ratio was evaluated using RT-qPCR, and compared to that of circulating lactate. At day 1, the *CDK1/CX3CR1* ratio had an AUROC of 0.74 (95% CI 0.64–0.83) to predict all-cause ICU mortality, not significantly different to that of lactate (AUROC 0.72, 95% CI 0.61–0.82). At day 3, the *CDK1/CX3CR1* ratio had an AUROC of 0.72 (95%CI 0.59–0.84) to predict all-cause ICU mortality, which was significantly higher than that of lactate alone (AUROC 0.67 [95% CI 0.54–0.79], *p*-value 0.008; Figure 2).

**Table 2:** Patient’s clinical and demographic characteristics of the independent validation cohort.

	Total (*n* = 140)	Survivors (*n* = 99)	Non-Survivors (*n* = 41)	*p*-value
Age, years	66 (57–77)	65 (55–77)	70 (63–79)	ns
Male gender	81 (58)	56 (57)	25 (61)	ns
Charlson score ≥ 1	107 (68)	73 (74)	34 (83)	ns
SAPSII score	61 (46–74)	57 (44–68)	73 (60–93)	<0.0001
Infection sites				ns
Pulmonary	73 (52)	52 (53)	21 (51)	
Abdominal	33 (24)	20 (20)	13 (32)	
Urinary tract	9 (6)	8 (8)	1 (2)	
Others	25 (18)	19 (19)	6 (15)	
Steroids	71 (51)	41 (41)	30 (73)	0.001
ICU mortality	41 (29)	0 (0)	41 (100)	

ns, non-significant; SAPS-II, Simplified Acute Physiology Score II; ICU, Intensive care unit. Medians and interquartile ranges [Q1-Q3] are shown for continuous variables or numbers and percentages are presented for categorical variables.

In the Sepsis-3 cohort, lactate values ≥2 mmol/L identify a group of patients with an approximately 40% overall mortality (1). We retrieved similar results in our validation cohort, with a 38% mortality in the group of 84 septic patients with a first lactate value ≥2 mmol/L. The mortality in patients with a lactate <2 mmol/L was 14%. In this cohort, specificity and sensitivity of the lactate performed at day 1 to predict all-cause ICU-mortality was 45 and 82%, respectively. At day 3, specificity and sensitivity were 74 and 44%, respectively. Of note, using the threshold value obtained after platform transfer, the specificity and sensitivity of *CDK1/CX3CR1* ratio at day 1 were 83 and 54%, respectively. At days 3, specificity and sensitivity of the *CDK1/CX3CR1* ratio were 91 and 44%, respectively. Although lactate harbor a higher sensitivity to predict ICU mortality, the specificity of the *CDK1/CX3CR1* ratio enables the identification of a group of patients with a higher risk of mortality at both time points (57% ICU mortality when performed at day 1 and 65% ICU mortality when performed at days 2–3; Supplementary Table 2).

### Prognostic and predictive enrichment in sepsis using lactate and the *CDK1/CX3CR1* ratio

**Figure 3:**
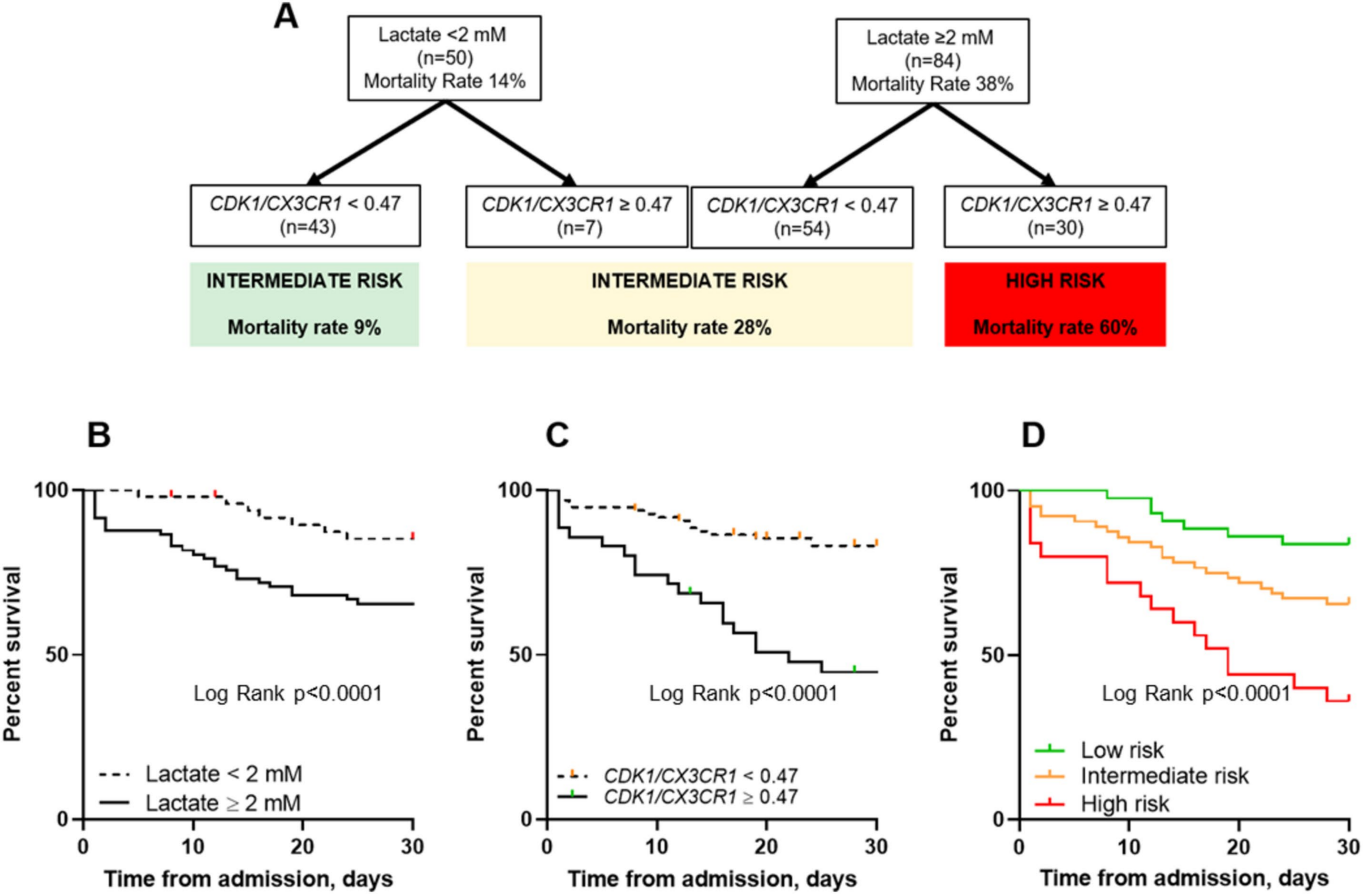
Decision algorithm combining lactate and the *CDK1/CX3CR1* ratio to predict all-cause ICU mortality at ICU admission **(A)**. Kaplan–Meier survival curves comparing all-cause ICU mortality (censored at day 30) using lactate with a cut-off value of 2 mM **(B)**, the *CDK1/CX3CR1* ratio with a cut-off value of 0.47 **(C)** and both classifiers **(D)**. The *p*-value for the log-rank test is provided for each plot: **(B)** *p* < 0.0001, **(C)** *p* < 0.0001, and **(D)** *p* < 0.0001. Additionally, pairwise comparisons of the survival curves based on risk stratification groups were also significant: Low versus Intermediate risk (*p* = 0.037), Intermediate versus High risk (*p* = 0.0076), and Low versus High risk (*p* < 0.0001). The same cut-off values for lactate and *CDK1/CX3CR1* ratio (2 mM and 0.47, respectively) were applied.

To evaluate the additive value of the CDK1/CX3CR1 ratio beyond the initial lactate-based stratification, we added the CDK1/CX3CR1 ratio to the initial severity grouping based on lactate recommendation levels (<2 or ≥ 2 mmol/L). When combining *CDK1/CX3CR1* ratio to lactate levels, we were able to identify 3 groups with increasing all-cause ICU mortality (Figure 3). At day 1, the group of patients with lactate ≥2 mmol/L and *CDK1/CX3CR1* ratio ≥ 0.47 had a 60% all-cause ICU mortality, compared to 38% lactate ≥2 stratification used alone. On the opposite, the group of patients with lactate <2 mmol/L and *CDK1/CX3CR1* ratio < 0.47 had a 9% all-cause ICU mortality (14% for lactate <2 mmol/L).

The group of patients in whom biomarkers were found to be discordant (i.e., lactate ≥2 mM and *CDK1/CX3CR1* ratio < 0.47, and lactate <2 mM and *CDK1/CX3CR1* ratio ≥ 0.47) had a 28% all-cause ICU mortality, defining an intermediate-risk group. This enrichment was also found at days 2–3, with a 67% all-cause ICU mortality in patients with lactate ≥2 mmol/L and *CDK1/CX3CR1* ratio ≥ 0.47 (Supplementary Figure 2). The main clinical characteristics of patients classified as low, intermediate, or high-risk using this enrichment strategy are summarized in Table 3.

**Table 3:** Patients’ characteristic according to low, intermediate or high-risk classification at day 1 and days 2–3.

Day 1				
	Low risk (*n* = 43)	Intermediate (*n* = 61)	High risk (*n* = 30)	*p*-value
Age, years	65 (55–77)	66 (57–79)	65 (62–75)	ns
Male gender	31 (72)	39 (61)	18 (67)	ns
Charlson score ≥ 1	32 (74)	50 (78)	22 (81)	ns
SOFA score	9 (7–10)	10 (8–12)	12 (9–14)	0.0002
SAPS-II score	55 (44–68)	62 (48–74)	68 (60–84)	0.0071
ICU mortality	4 (9)	18 (28)	17 (63)	<0.001
Days 2–3				
	Low risk (*n* = 59)	Intermediate (*n* = 22)	High risk (*n* = 12)	
Age, years	69 (58–78)	65 (50–75)	60 (56–64)	ns
Male gender	37 (63)	15 (68)	6 (50)	ns
Charlson score ≥ 1	42 (71)	17 (77)	10 (83)	ns
SOFA score	8 (5–10)	9 (7–13)	10 (7–15)	0.0364
SAPS-II score	60 (46–73)	58 (51–71)	68 (61–76)	ns
ICU mortality	11 (19)	6 (27)	8 (67)	0.003

ns, non-significant; SAPS-II, Simplified Acute Physiology Score II; SOFA score, sequential organ failure assessment score; ICU, Intensive care unit. Medians and interquartile ranges [Q1-Q3] are shown for continuous variables or numbers and percentages are presented for categorical variables.

## Discussion

In this study, we have identified a robust combination of two genes, *CDK1* and *CX3CR1*, whose ratio of expression has good performance to predict all-cause ICU mortality in septic patients, in a way that is consistent over the course of the first 3 days following ICU admission. The predictive performance of the *CDK1/CX3CR1* ratio is similar than that of lactate levels at day 1, and slightly higher when assessed at days 2–3. Finally, the *CDK1/CX3CR1* ratio can be used in combination with lactate levels for prognostic and predictive enrichment in sepsis patients.

Huckabee first suggested using elevated blood lactate levels as a measure of oxygen debt in hospitalized patients in 1961 (35). Since then, it has been widely accepted that lactate is a marker of anaerobic metabolism in patients with insufficient oxygen delivery in the setting of cardiovascular failure (2). However, several data failed to relate excess lactate to lack of oxygen and therefore suggest that mechanisms different from dysoxia can be linked to abnormal lactate levels in sepsis patients (36, 37). This multiplicity of underlying mechanisms may explain the low sensitivity of serum lactate levels to predict mortality in septic patients (38). While recent evidence suggests that lactate control could improve patient outcomes, its application as a biomarker to guide personalized therapy in sepsis remains limited by poor other operating characteristics, especially sensitivity and positive predictive values (4–6).

Consequently, identifying biomarkers that could guide resuscitation in sepsis remain of paramount importance (8, 39). However, owing to differences in clinical presentation, infectious source and uncertainty regarding the onset of sepsis, several studies have failed to validate the usefulness of biomarkers in independent cohorts (19, 40). In the majority of septic patients, the exact onset of sepsis remains unknown (41, 42). We therefore developed an innovative approach aiming to identify a limited set of transcriptomic biomarkers whose expression could reliably predict ICU mortality when sampled at different time points following ICU admission. This strategy has greatly limited the number of genes whose expression remains different between survivors and non-survivors across several time points, with only two genes downregulated and 39 genes upregulated in survivors vs. non-survivors. Interestingly, *CX3CR1* and *IL1B* were the two genes continuously downregulated in non-survivors vs. survivors, which is consistent with several previously published studies (43–46). Among the genes whose expression was upregulated in non-survivors vs. survivors, we identified *CDK1* as the main hub gene according to pathway analysis. This focus on hub genes was intended to limit collinearity and also to provide a better insight into the underlying molecular mechanisms involved in sepsis survival (47, 48). Interestingly, *CDK1* expression has previously been associated with mortality in sepsis (49), albeit in one unique report (50). *CDK1* is a central regulator involved in cell division processes and has been reported to be involved in hematological malignancies (51). Even if there is no experimental or clinical data investigating the role of this protein in sepsis or infection, its persistent elevation in non-survivors suggests a pivotal role in this setting.

We used the ratio of DEGs to build a combined biomarker, rather than by normalizing gene expression data by quantifying the expression of housekeeping genes. The rationale of this approach is to avoid potential biases related to the variability of expression of housekeeping genes, which are known to vary according to physiological or pathological conditions (52, 53). Moreover, the use of a ratio of genes whose expression varies in opposite directions (i.e., upregulated and downregulated) in non-survivors vs. survivors is useful to improve the predictive performance of the combined biomarker.

*CDK1* and *CX3CR1* genes expression were assessed using RT-qPCR, which is widely recognized as the most sensitive and specific method for quantifying mRNA (54), and then validated with a new independent cohort of 140 septic patients measured at two time points. Difficulties in translating the findings from transcriptomics studies to specific platforms used at the point-of-case for measuring gene expression (implementation platforms) have been a limitation to the usefulness of mRNA biomarkers signatures (55), which is why we set out to confirm the findings from the discovery cohort using RT-qPCR. This strategy allowed us to build a robust combination of transcriptomic biomarkers capable of identifying a group of septic patients at high risk of mortality throughout the first 72 h of ICU stay. Importantly, our findings were validated across cohorts that included patients with differing severity levels, ranging from sepsis to septic shock. This consistency supports the robustness of our methodology and highlights its potential applicability to a broad spectrum of septic patients. We decided to combine the *CDK1/CX3CR1* ratio with lactate to improve the stratification of patients at ICU admission. Indeed, the successive failures of numerous interventional trials in sepsis have highlighted the need to develop stratification strategies to identify patients who would benefit most from investigative therapies (56). When used in association with blood lactate levels, the *CDK1/CX3CR1* ratio provides improved enrichment performances, which suggests that it could be beneficial in this setting.

This study has several limitations. The retrospective design may induce uncontrolled selection and/or attrition bias. Additionally, the use of Sepsis-2 criteria to select patients, due to the data collection period, may limit the transferability of our findings to patients diagnosed according to Sepsis-3 criteria. Of note, the selection of patients still alive at days 3 after ICU admission in the discovery cohort may have limited the discovery of candidate genes. A second limitation of the study is the definition of the cut-off value of the *CDK1/CX3CR1* ratio in the transfer cohort. Finally, the small sample size of the transfer cohort limits the extrapolation of our results.

## Conclusion

We report a novel stratification strategy in septic patients that combines measurement of lactate levels and the expression of a combined mRNA biomarker by RT-qPCR, the *CDK1/CX3CR1* ratio. Importantly, the expression of *CDK1* and *CX3CR1* provides reliable prognostic information independently on when it is assessed over the three first days of ICU admission, suggesting that the CDK1/CX3CR1 could be an important biomarker in clinical practice.

## Data Availability

The datasets presented in this study can be found in online repositories. The names of the repository/repositories and accession number(s) can be found at: https://www.ncbi.nlm.nih.gov/, GSE95233; https://www.ncbi.nlm.nih.gov/, GSE57065.
