# Supplementary material for: Whole blood ratio of CDK1/CX3CR1 mRNA expression combined to lactate refines the prediction of ICU mortality in septic patients in the Sepsis-3 era: a proof-of-concept study
Source: Front Med (Lausanne). 2025 Jan 3;11:1445451. doi: 10.3389/fmed.2024.1445451 (PMC11739359; doi:10.3389/fmed.2024.1445451)
Supplement: Supplementary file 1 [file Data_Sheet_1.DOCX]

**SUPPLEMENTARY DATA**

**Supplementary table 1: Final 41 DEG common between the day 1 and days 2-3 analysis between survivors and non-survivors.**

| Lactate (threshold ≥2) |  |  | **Dead** | **Alive** |  | **ICU mortality** | Sensi | 0,82 |
| --- | --- | --- | --- | --- | --- | --- | --- | --- |
|  | **Day 1** | **High Risk** | **32** | **52** | **84** | **38%** | Speci | 0,45 |
|  |  | Low Risk | 7 | 43 | 50 | 14% | PPV | 0,38 |
|  |  |  | 39 | 95 | 134 |  | NPV | 0,86 |
|  |  |  | **Dead** | **Alive** |  |  | Sensi | 0,44 |
|  | **Days 2-3** | **High Risk** | **11** | **18** | **29** | **38%** | Speci | 0,74 |
|  |  | Low Risk | 14 | 50 | 64 | 22% | PPV | 0,38 |
|  |  |  | 25 | 68 | 93 |  | NPV | 0,78 |
| *CDK1/CX3CR1* (threshold ≥0,47) |  |  | **Dead** | **Alive** |  |  | Sensi | 0,54 |
|  | **Day 1** | **High Risk** | **21** | **16** | **37** | **57%** | Speci | 0,83 |
|  |  | Low Risk | 18 | 79 | 97 | 19% | PPV | 0,57 |
|  |  |  | 39 | 95 | 134 |  | NPV | 0,81 |
|  |  |  | **Dead** | **Alive** |  |  | Sensi | 0,44 |
|  | **Days 2-3** | **High Risk** | **11** | **6** | **17** | **65%** | Speci | 0,91 |
|  |  | Low Risk | 14 | 62 | 76 | 18% | PPV | 0,65 |
|  |  |  | 25 | 68 | 93 |  | NPV | 0,82 |

**Supplementary table 2. The contingency tables and predictive performance achieved for lactate and the *CDK1/CX3CR1* ratio when assessed at day 1 and days 2-3.**


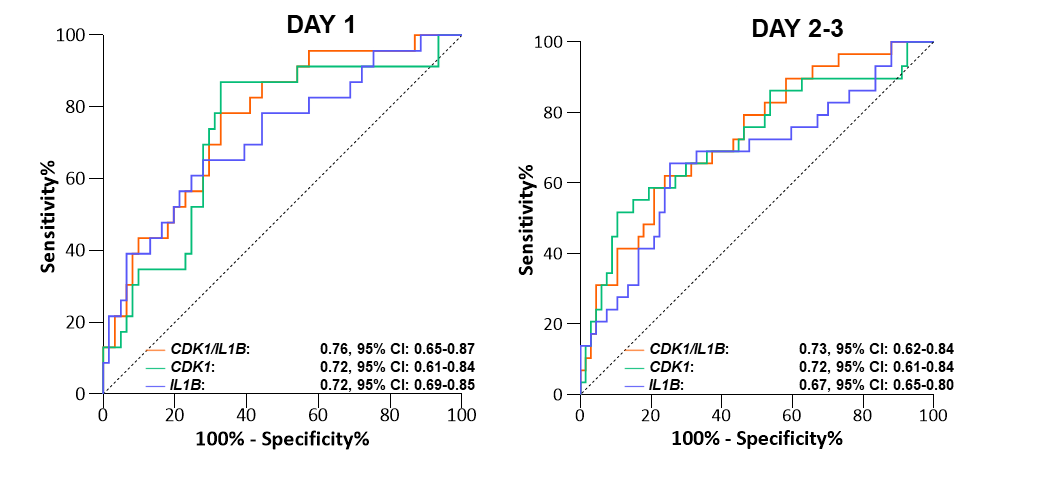


**Supplementary figure 1.** **Performance of the *CDK1* gene expression level, *IL1B* gene expression level, and *CDK1/IL1B* ratio to predict all-cause ICU mortality when assessed on (A) day 1 and (B) days 2-3 following ICU admission in septic patients (discovery cohort, n=100).**

Performance is given with area under the ROC curve (AUROC) and 95 % confidence interval.

**
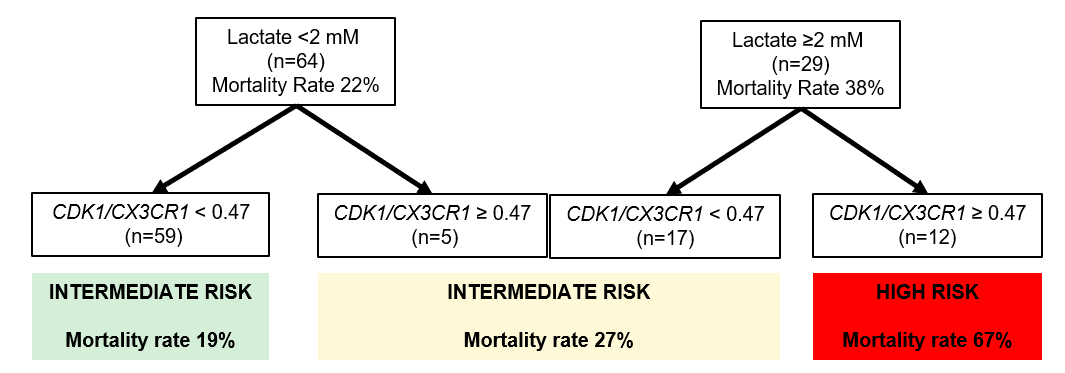
**

**Supplementary figure 2: Decision algorithm combining lactate and *CDK1/CX3CR1* ratio for risk assessment of ICU mortality at day 3.**
